# Supplementary material for: Cell-Free DNA Extracted from CSF for the Molecular Diagnosis of Pediatric Embryonal Brain Tumors
Source: Cancers (Basel). 2023 Jul 7;15(13):3532. doi: 10.3390/cancers15133532 (PMC10340330; doi:10.3390/cancers15133532)
Supplement: Supplementary file 1 [file cancers-15-03532-s001.zip › cancers-2460317-supplementary.pdf]

Supplemental Materials

Cell-Free DNA Extracted from CSF for the Molecular Diagnosis of Pediatric Embryonal Brain Tumors

Supplementary Materials

S1. Supplementary Methods

Molecular analysis of primary tumors

Clinical molecular diagnosis of primary pediatric EBT involved array-CGH, targeted sequencing and/or Nanostring profiling, according to the tumor entity. Molecular subtyping and panel sequencing with an in-house panel were performed as described previously [1–3].

Whole-exome sequencing (WES) of genomic DNA extracted from primary tumors, and paired germline genomic DNA, was performed in 18 and 6 patients, respectively. WES was performed following either an Agilent SureSelect Human All Exon v5 or a Roche Nimblegen SeqCap EZ Exome V3 library construction and capture, as reported previously [4].

cfDNA extraction and quantification

cfDNA was extracted from approximately 500 µl (5-6 droplets) of CSF using QIAamp Circulating Nucleic Acid Kit (Qiagen) with the Qiavac24s system, according to the manufacturers' recommendation. cfDNA concentration was measured using a Qubit fluorometric assay (Invitrogen) with the dsDNA HS (High Sensitivity) Assay Kit [5] . The total cfDNA concentration per mL of CSF was calculated. Its quality was defined based on a bioanalyzer agilent 2100 (Agilent) analysis using the High Sensitivity DNA chip.

Library construction and exome capture of cfDNA

cfDNA sequencing libraries were constructed without fragmentation using the Accel-NGS 2S Hyb DNA Library Kit for Illumina™ from Swift Biosciences® according to the manufacturer's protocol, modified to 9-cycle PCR, and with the addition of a size selection step by Ampure XP ™ beads to retain only the 160 bp fragments.

For whole exome capture, the Medexome Enrichment Kit (Nimblegen Roche Sequencing) was used according to the manufacturer's protocol. Paired-end (100 bp) Illumina™ WES was performed to an expected coverage of 100×.

Targeted sequencing panel design

For custom deep coverage targeted capture sequencing in order to infer expression based on the coverage of sequencing around the TSS, a panel was designed with Nimbledesign tools to cover EBT-relevant genes and their TSS. One hundred and twelve genes which contribute to the genetic classification of embryonic tumors such as *MYC* (in MB and ATRT MYC subtype), *MYCN* (in MB and ATRT SHH subtype), *TYR* (in ATRT TYR subtype) or *JAK3* are used for a diagnostic Nanostring panel design at Institut Curie[6–8]. The regions (+/- 1 kb) surrounding the TSS of these genes were included in the design for a panel to analyze CSF cfDNA. In addition, the coding sequences of six genes (*CTNNB1*, *PTCH1*, *SMARCB1*, *SMARCA4*, *SMO*, and *SUFU*) were added to enable the use for this panel for detection of driver mutation in these tumors, as well as five hotspots mutations of gliomas (*H3F3A*_K27M, *HIST1H3B*_K27M, *BRAF*_V600E, *IDH1*_R132C and *IDH2*_R172K) [9,10]. Forty-four SNPs were also added for identity monitoring. For the prediction of expression, eight TSS were selected as controls, including four ubiquitously expressed genes with strong or moderate expression such as *ACTB*, *B2M*, *GAPDH* or *SDHA431*. Two

genes not expressed in the central nervous system (*BAGE4* and *ACPT*), and two genes expressed in the CNS (*SLC32A1* and *CNTNAP2*) were selected from the Protein Atlas database [11,12] This panel design is referred to as the targeted sequencing TSS panel.

This targeted sequencing TSS panel was used either directly on cfDNA or for a second capture step on previously constructed and captured WES libraries. Following a double capture procedure, first of the whole exome followed by the targeted capture, target sequencing (PE 100 × 100 bp) was performed on all CSF samples on the Miseq V3 or Novaseq SP aiming for a theoretical coverage of 500x.

Bioinformatics analysis:

Copy number and SNV analysis

The WES and target sequencing raw reads were mapped to the reference human genome assembly GRCh37/hg19 using BWA v0.7.15, with default parameters. The data were preprocessed for variant discovery according to GATK Best Practices for WES, and without duplicates removal for target sequencing.

Coverage scores were computed and normalized using deepTools bamCoverage function with CPM method respectively[13].

Variants were called with GATK 4.1.7.0 Mutect2 using Joint-Calling method. A minimum mapping and base quality of 20 was applied in each calling method. A panel of 200 normal germline samples and a germline ressource from gnomAD (somatic-b37_af-only-gnomad.raw.sites.vcf) was used to filter germline variants. The variants were annotated with SnpEff 5.0 (dbNSFP2.9.3, COSMIC v92, gnomAD r.2.1.1, clinvar_20200824).

Only SNVs with a Moderate or High predicted impact were kept. Variants with a population prevalence of greater than 1/2,000, a coverage less than 20, a number of reads that match the variant fewer than 10, and variants that have no cosmicID or that are not predicted to be deleterious by at least 3/9 databases were filtered out. IGV v2.4.14 was used to generate SNV pictures and the IGVs were verified individually by visual inspection.

For targeted sequecing analysis, only results of calling by Mutect2 were considered given the small targeted regions. Unlike WES data analysis, no additional filter was applied. Copy number analyses were performed with snp-pileup_0.5.14 and FACETS v0.5.11 applying a threshold of 20 for mapping and base quality. The option "unmatch" was used for patients without a paired normal sample.

The ctDNA fraction in cfDNA was predicted using the Facets tool by using a matched normal sample whenever available and unmatched normal sample when no matched normal sample was available [14].

Sequencing across the TSS to infer expression profiles

The bigwig files containing the coverage scores representing the number of reads per bin size of 1 were generated with the option BAM coverage using BAM files[13] . Only fragments sized between 120pb and 180pb were kept. The MNase option was used to determine nucleosome position and extension of reads to fragment size was allowed. Only reads with mapping quality higher than 20 were kept. All duplicates were removed and the CPM (Counts Per Million mapped reads) method was used to normalize the number of reads per bin. Matrices with mean score per genomic region (-300/-1,000 bp downstream of the TSS and +300/+1,000 bp upstream of the TSS) were computed with the option computeMatrix and used as input for the option PlotProfile that allows to visualise the average read coverages over the genomic regions and also to the option plotHeatmap that allows the visualisation of the read coverages for genomic regions.

The window capture of the TSSs had a size of 2 kb around the TSS (-1 kb downstream and +1 kb upstream of the TSS) to include the nucleosome depleted region (NDR) and to observe the TSS footprint at this position. K-means clustering using deeptools, with the number of clusters set to 2, was used to classify genes based on the coverage across the TSS.

References

1. Euskirchen P, Bielle F, Labreche K, Kloosterman WP, Rosenberg S, Daniau M, Schmitt C, Masliah-Planchon J, Bourdeaut F, Dehais C *et al*: **Same-day genomic and epigenomic diagnosis of brain tumors using real-time nanopore sequencing**. *Acta Neuropathol* 2017, **134**(5):691-703.
2. Passeri T, Dahmani A, Masliah-Planchon J, Naguez A, Michou M, El Botty R, Vacher S, Bouarich R, Nicolas A, Polivka M *et al*: **Dramatic In Vivo Efficacy of the EZH2-Inhibitor Tazemetostat in PBRM1-Mutated Human Chordoma Xenograft**. *Cancers (Basel)* 2022, **14**(6).
3. Richer W, Masliah-Planchon J, Clement N, Jimenez I, Maillot L, Gentien D, Albaud B, Chemlali W, Galant C, Larousserie F *et al*: **Embryonic signature distinguishes pediatric and adult rhabdoid tumors from other SMARCB1-deficient cancers**. *Oncotarget* 2017, **8**(21):34245-34257.
4. Berlanga P, Pierron G, Lacroix L, Chicard M, Adam de Beaumais T, Marchais A, Harttrampf AC, Iddir Y, Larive A, Soriano Fernandez A *et al*: **The European MAPPYACTS Trial: Precision Medicine Program in Pediatric and Adolescent Patients with Recurrent Malignancies**. *Cancer Discov* 2022, **12**(5):1266-1281.
5. Chicard M, Colmet-Daage L, Clement N, Danzon A, Bohec M, Bernard V, Baulande S, Bellini A, Deveau P, Pierron G *et al*: **Whole-Exome Sequencing of Cell-Free DNA Reveals Temporo-spatial Heterogeneity and Identifies Treatment-Resistant Clones in Neuroblastoma**. *Clin Cancer Res* 2018, **24**(4):939-949.
6. Sturm D, Orr BA, Toprak UH, Hovestadt V, Jones DTW, Capper D, Sill M, Buchhalter I, Northcott PA, Leis I *et al*: **New Brain Tumor Entities Emerge from Molecular Classification of CNS-PNETs**. *Cell* 2016, **164**(5):1060-1072.
7. Johann PD, Erkek S, Zapatka M, Kerl K, Buchhalter I, Hovestadt V, Jones DTW, Sturm D, Hermann C, Segura Wang M *et al*: **Atypical Teratoid/Rhabdoid Tumors Are Comprised of Three Epigenetic Subgroups with Distinct Enhancer Landscapes**. *Cancer Cell* 2016, **29**(3):379-393.
8. Ramaswamy V, Remke M, Bouffet E, Faria CC, Perreault S, Cho YJ, Shih DJ, Luu B, Dubuc AM, Northcott PA *et al*: **Recurrence patterns across medulloblastoma subgroups: an integrated clinical and molecular analysis**. *Lancet Oncol* 2013, **14**(12):1200-1207.
9. Behling F, Schittenhelm J: **Oncogenic BRAF Alterations and Their Role in Brain Tumors**. *Cancers (Basel)* 2019, **11**(6).
10. Wu G, Diaz AK, Paugh BS, Rankin SL, Ju B, Li Y, Zhu X, Qu C, Chen X, Zhang J *et al*: **The genomic landscape of diffuse intrinsic pontine glioma and pediatric non-brainstem high-grade glioma**. *Nat Genet* 2014, **46**(5):444-450.
11. Consortium GT: **The Genotype-Tissue Expression (GTEx) project**. *Nat Genet* 2013, **45**(6):580-585.
12. Lizio M, Harshbarger J, Shimoji H, Severin J, Kasukawa T, Sahin S, Abugessaisa I, Fukuda S, Hori F, Ishikawa-Kato S *et al*: **Gateways to the FANTOM5 promoter level mammalian expression atlas**. *Genome Biol* 2015, **16**(1):22.
13. Ramirez F, Ryan DP, Gruning B, Bhardwaj V, Kilpert F, Richter AS, Heyne S, Dundar F, Manke T: **deepTools2: a next generation web server for deep-sequencing data analysis**. *Nucleic Acids Res* 2016, **44**(W1):W160-165.
14. Shen R, Seshan VE. **FACETS: allele-specific copy number and clonal heterogeneity analysis tool for high-throughput DNA sequencing**. *Nucleic Acids Res* 2016 44(16):e131

2. Supplementary Figures

Supplementary Figure S1

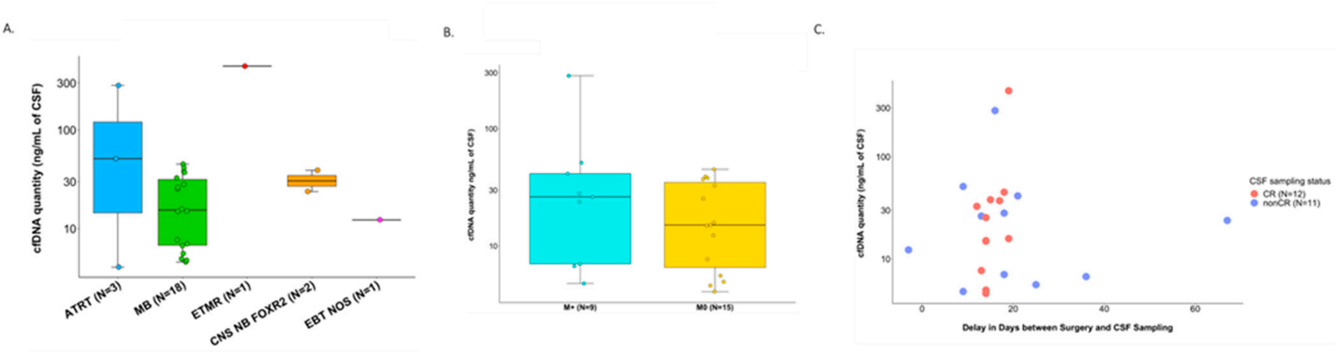

Supplementary Figure S1: cfDNA concentrations extracted from 5 – 6 droplets CSF, quantified per mL of CSF. A. cfDNA concentrations depending on the disease type. B. cfDNA concentrations depending on disease status. C. cfDNA concentrations depending on time between surgery of the primary tumor and CSF collection. Disease status at time of CSF sampling: CR, complete remission (red); nonCR, not in complete remission (blue). No correlation between cfDNA concentration and disease entity, disease status (localised versus metastatic), or time between surgery and CSF collection could be observed.

Supplementary Figure S2

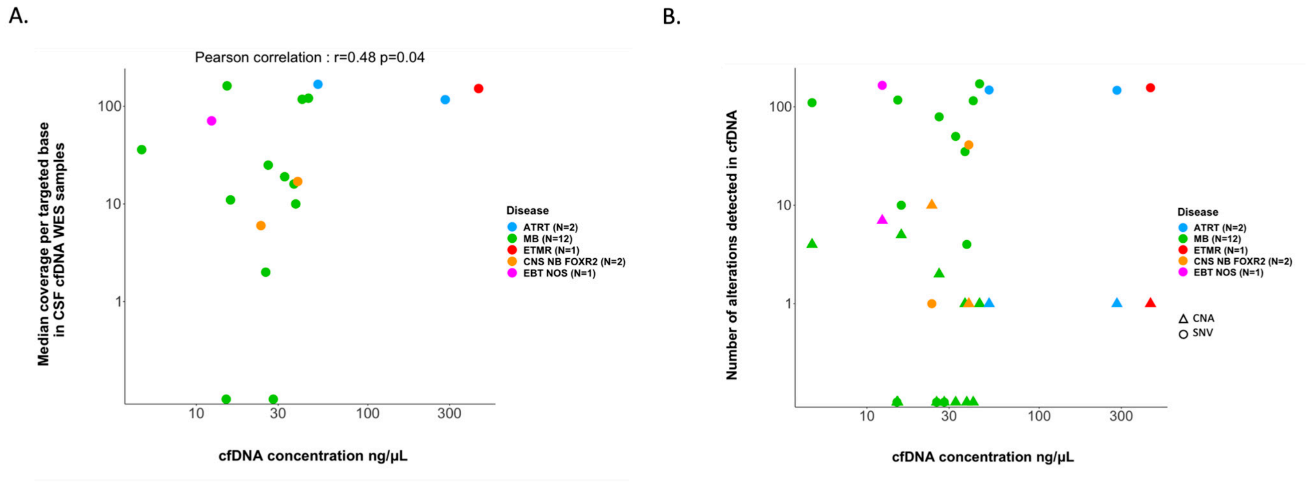

Supplementary Figure S2: A. Median coverage per targeted base in CSF WES sample according to the cfDNA concentration. Pearson test shows significant correlation (r=0.48, p=0.04). B. Number of CNAs and SNVs according to the cfDNA concentration. Pearson test shows no significant correlation (r=0.2, p=0.1).

Supplementary Figure S3

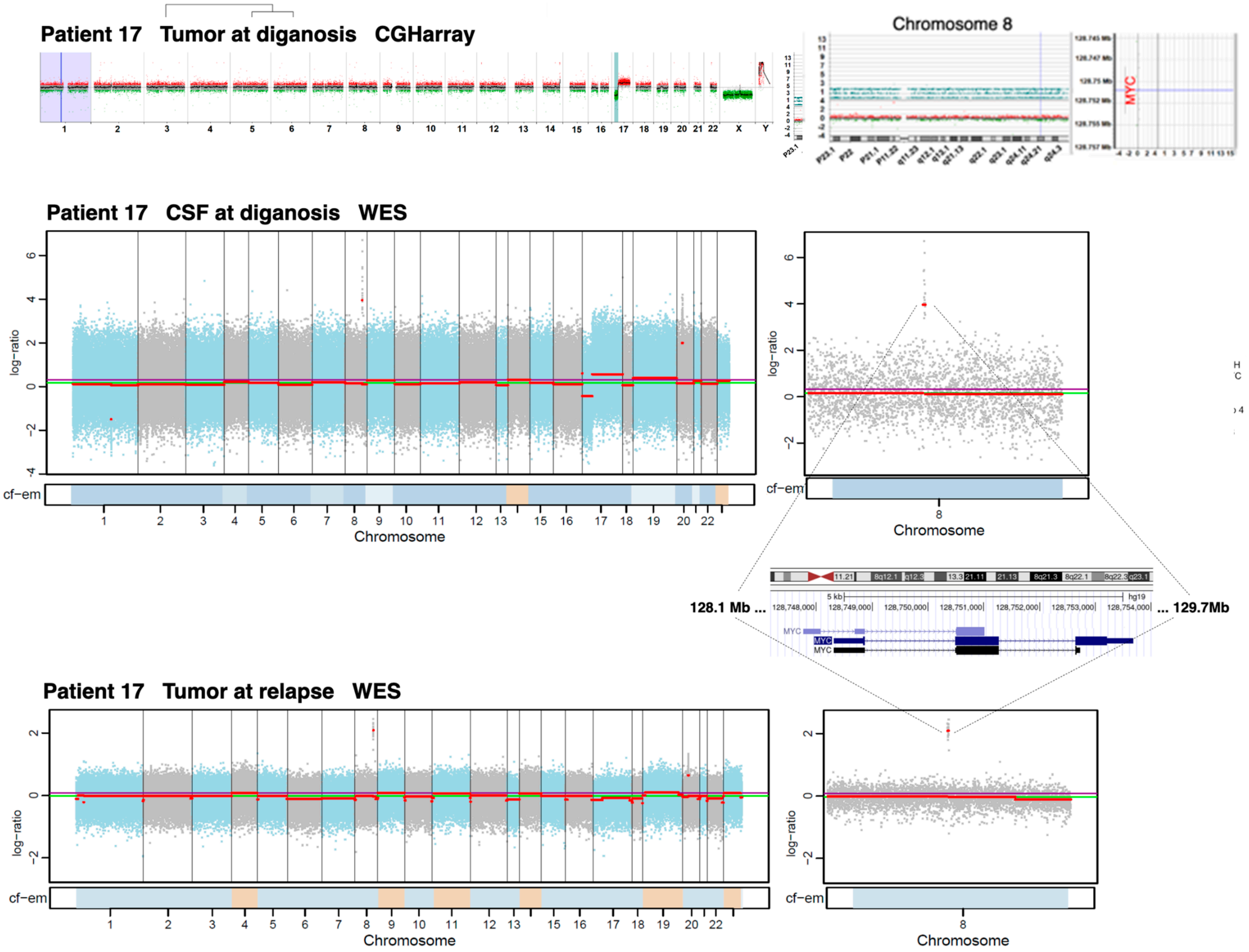

Supplementary Figure S3: CSF cfDNA copy number analysis reveals a *MYC* amplification not seen in the analysis of the primary tumor at diagnosis, but in the tumor at the time of relapse (patient 17, with a diagnosis of MB). A. aCGH on DNA extracted from the primary tumor at diagnosis, showing a dynamic copy number profile, with loss of chr 17p, and gain of chr 17q, but no *MYC* amplification. B. Copy number profile by WES on CSF cfDNA reveals a *MYC* amplification, in addition to 17p loss/17q gain. C. At relapse, WES on the recurrent tumor reveals *MYC* amplification. These findings indicate clonal heterogeneity, with the *MYC* amplification most likely not present in the cells analyzed in the primary tumor sample at diagnosis, but contributing to the relapse.

Supplementary Figure S4

Supplementary Figure S4: Clustering on the total 112 NDR regions of the capture in the seven samples for which the k-means clustering of genes into two groups was informative. Hierarchical clustering was performed on NDR and on samples.

Supplementary Table S1a: Clinical and biological characteristics of 25 pediatric patients with embryonal brain tumors. Detailed clinical information, and follow up. Biological analyses performed on primary tumor and CSF cfDNA are indicated. Abbreviations : m, months; d, days; M, Metastasis; NA , not applicable; MD , missing data; FU, follow-up; CR, complete remission; PR , partial remission; PD, progressive disease; DOD, dead of disease

Patient Number	Age at diagnosis (m)	Pathological Diagnosis	Molecular group	Localisation	M	Tumor molecular analysis	CSF molecular analysis	Surgery results	CSF Cytological examination	Delay - surgery to CSF sampling (d)	status at CSF sampling	Treatment/ protocol	Occurrence of relapse	Disease status at last FU	FU duration (m)
1	93,3	ATRT	ATRT_SHH	posterior fossa	M+	at diagnosis	at diagnosis	incomplete resection	negative	16	nonCR	ATRT DFCI	no	CR	35,23
2	15,9	ATRT	ATRT_MYC	supra-tentorial, frontal	M+	at diagnosis	at diagnosis	incomplete resection	negative	9	nonCR	EU-RHAB, then MEMMAT-like	no	PR	31,80
3	6,2	ATRT	not done/not contributive	posterior fossa	M0	at diagnosis	at diagnosis	incomplete resection	MD	MD	nonCR	ATRT09	no	DOD	8,37
4	65,1	Medulloblastoma	Group 4	posterior fossa	M3	at diagnosis	at diagnosis	incomplete resection	negative	18	nonCR	PNETHR+5	no	CR	123,37
5	105,3	Medulloblastoma	WNT	posterior fossa	M0	at diagnosis	at diagnosis	complete resection	negative	18	CR	M-SFOP 2007	no	CR	125,07
6	109,0	Medulloblastoma	not done/not contributive	posterior fossa	M0	at diagnosis	at diagnosis	complete resection	negative	15	CR	PNET4	no	CR	100,00
7	204,8	Medulloblastoma	not done/not contributive	posterior fossa	M0	at diagnosis	at diagnosis	complete resection	negative	12	CR	hybride	no	CR	100,00
8	35,5	CNS NB FOXR2	not done/not contributive	supratentorial, right temporoparietal region	M3	at diagnosis	at diagnosis	incomplete resection	after chemotherapy : 2 atypical cells	67	nonCR	hybride	yes	DOD	13,53
9	141,3	Medulloblastoma	Group 4	posterior fossa	M0	at diagnosis	at diagnosis	complete resection	negative	14	CR	MSFOP 2007	no	CR	86,43
10	59,3	Medulloblastoma	NonWNT/ nonSHH	posterior fossa	M0	at diagnosis	at diagnosis	complete resection	negative	19	CR	PNETHR+5	no	CR	97,93

11	347,5	Medulloblastoma	SHH	posterior fossa	M0	at diagnosis	at diagnosis	complete resection	negative	14	CR	RSMA	no	CR	75,13
12	84,9	Medulloblastoma	WNT	posterior fossa	M0	at diagnosis	at diagnosis	complete resection	negative	17	CR	PNET5-like	no	CR	65,43
13	122,6	Medulloblastoma	NonWnt/ NonSHH	posterior fossa	M+	at diagnosis	at diagnosis	incomplete resection	positive	13	nonCR	PNET HR+5	no	CR	66,23
14	410,5	Medulloblastoma	not done/not contributive	posterior fossa	M+	at diagnosis	at diagnosis	incomplete resection	suspect	21	nonCR	PNET HR+5	no	CR	62,57
15	47,1	Medulloblastoma	Group 3	posterior fossa	M+	at diagnosis	at diagnosis	incomplete resection	negative	18	nonCR	HRMB-5	yes	PD	62,23
16	186,8	Medulloblastoma	Group 3	posterior fossa	M0	at diagnosis	at diagnosis	complete resection	negative	14	CR	PNET5	no	CR	52,20
17	82,5	Medulloblastoma	Group4	posterior fossa	M+	at relapse	at diagnosis	incomplete resection	positive	9	nonCR	PNET HR+5	yes	DOD	27,27
18	140,5	Medulloblastoma	Group 4	posterior fossa	M0	at diagnosis	at diagnosis	complete resection	negative	14	CR	PNET5	no	CR	32,50
19	111,8	Medulloblastoma	Group 4	posterior fossa	M0	at diagnosis	at diagnosis	complete resection	negative	13	CR	PNET5	no	CR	30,27
20	140,0	Medulloblastoma	WNT	posterior fossa	M0	at diagnosis	at diagnosis	complete resection	negative	14	CR	PNET5	no	CR	34,70
21	172,3	Medulloblastoma	Group 4	posterior fossa	M0	at diagnosis	at diagnosis	incomplete resection	negative	25	nonCR	PNET HR+5	yes	DOD	17,90
22	58,3	Medulloblastoma	Group 3	posterior fossa	M+	at diagnosis	at diagnosis	complete resection	positive	36	nonCR	PNET HR+5	no	CR	45,83
23	40,1	ETMR	ETMR	MD	MD	at diagnosis	at diagnosis	complete resection	negative	19	CR	PNET HR-5	yes	DOD	19,77
24	63,6	CNS NB FOXR2	NB-FOXR2	posterior fossa	M0	at relapse	at relapse	complete resection	MD	MD		other	yes		
25	39,9	Embryonic brain tumor NOS	Malignant plexus choroid tumor	supratentorial	M0	at diagnosis	at diagnosis	complete resection (2nd surgery)	negative	-3	nonCR	PNET HR+5	yes	DOD	15,93

Supplementary Table S1b : Results of CSF cfDNA analysis

Abbreviations PT, primary tumor; GL, germline analysis A, Total number of WES SNV in tumor; B, Number of WES SNVs common to tumor and CSF cfDNA; C, Number of WES SNV in tumor but not in CSF cfDNA; D, Number of WES SNV in CSF cfDNA but not in tumor; E, Number of SNV in tumor WES not covered in CSF cfDNA WES; F, Number of SNV in CSF cfDNA WES not covered in tumor WES; G, Total number of SNV in CSF cfDNA (WES); neccc, not enough coverage in CSF cfDNA. The ctDNA fraction in cfDNA was predicted using the Facets tool [14] by using a matched normal sample whenever available * and unmatched normal sample when no matched normal sample was available.

Patient number	CSF cfDNA concentration (ng/ml)	ctDNA fraction in cfDNA	GL	PT data type	CSF cfDNA : data type	CNA PT analysis : contributivity	CNA CSF cfDNA: contributivity	SNV PT : contributivity	SNV CSF cfDNA : contributivity	A	B	C	D	E	F	G	CSFcfDNA target seq TSS: Deeptools Kmeans	CSF cfDNA target seq TSS: Mean coverage
1	282.7	0.23*	Yes	WES	WES/ target seq	Yes	Yes	Yes	Yes	153	134	15	4	4	9	147	informative	311
2	51.2	0.3*	Yes	WES	WES/ target seq	Yes	Yes	Yes	Yes	156	143	11	5	2	0	148	informative	628
3	4.08	NA	NA	panel	target seq	NA	NA	Yes	NA	NA	NA	NA	NA	NA	NA	NA	non-informative	7
4	28.1	<0.15	NA	WES	WES	Yes	No	Yes	No	161	neccc	neccc	neccc	161	0	0	NA	
5	45.06	<0.15	NA	WES	WES/ target seq	Yes	Yes	Yes	Yes	183	160	22	11	1	0	171	informative	331
6	37.98	<0.15	NA	WES	WES	Yes	No	Yes	Yes	145	4	8	0	133	0	4	NA	
7	32.73	<0.15	NA	WES	WES	Yes	No	Yes	Yes	141	47	31	3	63	0	50	NA	
8	23.8	<0.15	NA	WES	WES	Yes	Yes	Yes	Yes	141	1	1	0	139	0	1	NA	
9	25.38	<0.15	NA	WES	WES	Yes	No	Yes	No	136	neccc	neccc	neccc	136	0	0	NA	
10	15.82	<0.15	NA	WES	WES	Yes	Yes	Yes	Yes	157	10	11	0	136	0	10	NA	
11	14.93	<0.15	NA	WES	WES	Yes	No	Yes	No	225	neccc	neccc	neccc	225	0	0	NA	
12	37.1	<0.15	NA	WES	WES	Yes	Yes	Yes	Yes	134	35	14	0	85	0	35	NA	
13	26.25	<0.15	NA	WES	WES	Yes	Yes	Yes	Yes	145	78	31	1	36	0	79	NA	
14	41.42	0.48	NA	WES	WES/ target seq	Yes	No	Yes	Yes	170	107	62	6	1	2	115	informative	218
15	7.04	NA	NA	panel	target seq	No	NA	Yes	NA	NA	NA	NA	NA	NA	NA	NA	non-informative	4
16	4.6	NA	NA	panel	target seq	Yes	NA	Yes	NA	NA	NA	NA	NA	NA	NA	NA	non-informative	16
17	4.8	0.37*	Yes	WES	WES/ target seq	Yes	Yes	Yes	Yes	122	99	13	6	10	5	110	non-informative	142
18	15.1	<0.15*	Yes	WES/	WES/	Yes	No	Yes	Yes	108	100	8	15	0	2	117	informative	478

				panel	target seq													
19	7.7	NA	NA	panel	target seq	Yes	NA	Yes	NA	NA	NA	NA	NA	NA	NA	NA	non-informative	13
20	4.96	NA	NA	panel	target seq	Yes	NA	Yes	NA	NA	NA	NA	NA	NA	NA	NA	non-informative	10
21	5.6	NA	NA	panel	target seq	Yes	NA	Yes	NA	NA	NA	NA	NA	NA	NA	NA	non-informative	19
22	6.72	NA	NA	panel	target seq	Yes	NA	Yes	NA	NA	NA	NA	NA	NA	NA	NA	non-informative	5
23	442.75	NA	NA	WES	WES/ target seq	Yes	Yes	Yes	Yes	149	131	16	25	2	0	156	informative	594
24	39.03	0.25*	Yes	WES	WES	Yes	Yes	Yes	Yes	132	37	20	1	75	3	41	NA	
25	12.27	0.86*	Yes	WES	WES/t arget seq	Yes	Yes	Yes	Yes	165	153	10	4	2	8	165	informative	142

Supplementary Table S2: Quality control, WES. For WES the sequencing coverage and quality statistics for each sample are summarized in Supplementary Table 2. The reference genome assembly GRCh37 was used to map the reads.

Sample ID	Total number of sequenced reads	Total number of uniquely mapped non-duplicate reads	Total number of covered bases	Median coverage (and range) per base	Percentage of targeted bases with coverage ≥ 10
Patient1_Tumor	260033903	220615451	12183244282	214 (0-9389)	97.2 %
Patient2_Tumor	296520478	171159112	12260403193	228 (0-8987)	99.9 %
Patient4_Tumor	136881215	117621447	8103125357	144 (0-6439)	99.7 %
Patient5_Tumor	117855631	92992148	6549460078	103 (0-3204)	99.5 %
Patient6_Tumor	158413365	138393034	9699137817	182 (0-4357)	99.5 %
Patient7_Tumor	138778280	116335272	8081268705	148 (0-5257)	99.6 %
Patient8_Tumor	97630544	82598599	5732707808	108 (0-2775)	99.1 %
Patient9_Tumor	119476092	91435973	6419339389	118 (0-5357)	99.6 %
Patient10_Tumor	99334977	84144656	5909401786	105 (0-5727)	99.3 %
Patient11_Tumor	143762885	118218123	8390405723	154 (0-3756)	99.5 %
Patient12_Tumor	149866333	80040781	5582712151	104 (0-3576)	99.1 %
Patient13_Tumor	127969900	66563229	4699904836	88 (0-4808)	99.1 %
Patient14_Tumor	143967988	120331916	8747134118	163 (0-7814)	99.4 %
Patient17_Tumor	277249764	238603928	12961800779	230 (0-11851)	97.3 %
Patient18_Tumor	216520237	61476520	4009446709	81 (0-3208)	99.8 %
Patient23_Tumor	159888987	134820579	9538820722	182 (0-4092)	99.4 %
Patient24_Tumor	203466454	179035447	9939727278	182 (0-12856)	95.4 %
Patient25_Tumor	181915814	153363552	9030624979	153 (0-16933)	96.6 %
Patient1_CSF	206811704	94804735	5921092816	117 (0-3476)	99.9 %
Patient2_CSF	204757080	127305642	8787334419	168 (0-5957)	99.9 %

Patient4_CSF	11293457	824750	39196230	0 (0-73)	0.2 %
Patient5_CSF	117231304	95355287	6494217020	121 (0-4127)	99.4 %
Patient6_CSF	148514872	11102521	534327825	10 (0-927)	52.8 %
Patient7_CSF	280311962	20418395	975031735	19 (0-1564)	89.1 %
Patient8_CSF	106737549	7875548	350336110	6 (0-613)	26.5 %
Patient9_CSF	34538873	2605394	105880859	2 (0-186)	1.1 %
Patient10_CSF	164484994	12300090	579074976	11 (0-1579)	60.8 %
Patient11_CSF	5562952	453824	18225913	0 (0-302)	0.1 %
Patient12_CSF	80758010	13130234	813768394	16 (0-912)	83.4 %
Patient13_CSF	107550453	19789478	1252420522	25 (0-2028)	95.6 %
Patient14_CSF	124273645	106480569	6319973508	118 (0-6919)	99.4 %
Patient17_CSF	242702066	30637456	1815483492	36 (0-2804)	98.9 %
Patient18_CSF	278477501	128808664	8837252109	162 (0-4452)	99.9 %
Patient23_CSF	144374376	121295553	8043794344	152 (0-3433)	98.9 %
Patient24_CSF	253242260	19031640	900290161	17 (0-1718)	83.5 %
Patient25_CSF	222242005	56674264	3677216188	71 (0-5909)	99.6 %

Supplementary Table S3: Quality control, Targeted sequencing panel. For targeted sequencing panel analysis, the sequencing coverage and quality statistics for each sample are summarized in Supplementary Table 3. The reference genome assembly GRCh37 was used to map the reads.

Sample ID	Total number of sequenced reads	Total number of uniquely mapped non-duplicate reads	Total number of covered targeted bases	Median coverage (and range) per targeted base	Percentage of targeted bases with coverage ≥ 200
Patient1_CSF	109051890	1631458	16054877	330 (7-960)	93.1 %
Patient2_CSF	109409168	2864393	35833589	782 (4-1978)	96.3 %
Patient3_CSF	7687154	37040	416294	7 (0-278)	0.2 %
Patient5_CSF	4207467	1445105	20741148	447 (0-812)	91.7 %
Patient14_CSF	3986259	971701	13618344	297 (0-514)	84.8 %
Patient15_CSF	3998057	24149	262958	5 (0-46)	0 %
Patient16_CSF	32003917	86323	871059	16 (0-279)	0.2 %
Patient17_CSF	198291143	697823	6337765	121 (0-2115)	7.9 %
Patient18_CSF	172295141	2471517	25269567	524 (1-2165)	96.4 %
Patient19_CSF	16923700	74033	741372	14 (0-273)	0.2 %
Patient20_CSF	12390628	49882	536426	10 (0-149)	0 %
Patient21_CSF	40811654	109722	1113900	20 (0-483)	0.4 %
Patient22_CSF	3319548	22915	283564	5 (0-291)	0.1 %
Patient23_CSF	4732199	2466168	39428138	854 (0-1952)	92.8 %
Patient25_CSF	129290153	658545	6911980	133 (1-1278)	13.4 %
